# Purulent Pericarditis and Mycotic Aortic Arch Aneurysm Caused by Salmonella Typhimurium: A Rare Case in an Immunocompromised Patient

**DOI:** 10.7759/cureus.100198

**Published:** 2025-12-27

**Authors:** Marta Machado, Catarina Negrão, Margarida Mourato, João Tiago Serra

**Affiliations:** 1 Department of Internal Medicine II, Hospital Professor Doutor Fernando Fonseca, Amadora, PRT; 2 Department of Internal Medicine III, Hospital Professor Doutor Fernando Fonseca, Amadora, PRT

**Keywords:** cardiac tamponade, mycotic aneurysm, pericarditis, purulent pericarditis, salmonella typhimurium

## Abstract

Purulent pericarditis is a rare but life-threatening condition, and *Salmonella* Typhimuriumis an uncommon pathogen. Its simultaneous occurrence with a mycotic aortic aneurysm is exceptionally rare and has been documented only in a very limited number of cases. We describe the case of a 64-year-old woman with long-standing systemic lupus erythematosus managed with chronic corticosteroid therapy who presented with fever and pleuritic chest pain. Laboratory tests showed elevated inflammatory markers, and the electrocardiogram demonstrated diffuse ST-segment elevation with PR-segment depression. She was diagnosed with acute pericarditis and discharged with non-steroidal anti-inflammatory drugs. Nine days later, she returned with recurrent fever, chest pain, dyspnea, and orthopnea. Computed tomography revealed a large pericardial effusion and a saccular aneurysm of the aortic arch. Given the presence of arterial hypotension and echocardiographic signs of cardiac tamponade, urgent pericardiocentesis was performed with drainage of purulent fluid, and intravenous ceftriaxone was initiated on the first day. Pericardial fluid culture isolated *Salmonella *Typhimurium. Despite clinical and laboratory improvement, serial echocardiograms demonstrated progressive features of constrictive pericarditis. Due to the evolving constrictive pericarditis and the concomitant presence of a mycotic aortic arch aneurysm at risk of rupture, complete pericardiectomy and aortic arch replacement were performed.

This case illustrates an exceptionally rare presentation of invasive non-typhoidal *Salmonella* infection manifesting exclusively with extra-intestinal symptoms and complications (purulent pericarditis and a mycotic aortic aneurysm). Early recognition, prompt pericardial drainage, targeted antibiotic therapy, and timely surgical intervention were essential for a favorable outcome, especially in an immunosuppressed patient.

## Introduction

Pericarditis is an inflammatory syndrome of the pericardium that may be associated with pericardial effusion. Acute pericarditis, defined as lasting less than four weeks, is a relatively common condition that predominantly affects younger adult males, with an estimated incidence of 3-32 cases per 100,000 person-years [1]. In high-income countries, most cases are idiopathic or viral, whereas tuberculosis predominates in endemic regions [1,2].

Chest pain is the main clinical feature and is usually sharp, retrosternal, pleuritic, and positional. Less frequent symptoms include dyspnea, fever, and fatigue [1,2]. A definitive diagnosis requires the presence of typical symptoms along with at least one supporting criterion, such as a pericardial friction rub, characteristic electrocardiographic changes, elevated inflammatory markers, pericardial effusion, or supportive findings on cardiac magnetic resonance imaging [1]. 

The two most serious complications of pericarditis are cardiac tamponade and constrictive pericarditis, which occur more frequently in bacterial pericarditis, particularly in the setting of purulent disease [3-5]. Unlike idiopathic or viral pericarditis, purulent pericarditis carries a markedly worse outcome, with reported mortality rates of 15-40% even with appropriate treatment and a substantially higher risk of cardiac tamponade (20-30%) and constrictive pericarditis, highlighting the importance of early evaluation and treatment to prevent severe complications [1-3,5]. Bacterial or purulent pericarditis should be suspected in patients presenting with acute pericarditis accompanied by fever and a marked inflammatory response, particularly in immunocompromised patients.

Purulent pericarditis is an uncommon but life-threatening infection that may arise by contiguous intrathoracic spread, hematogenous seeding, or direct inoculation (trauma or postsurgical) [5]. In developed countries, *Staphylococcus *spp. and *Streptococcus *spp. are the most commonly isolated pathogens [4].

Mycotic aneurysms represent another rare extraintestinal manifestation of *Salmonella* infection, arising from hematogenous dissemination during bacteremia with subsequent invasion of compromised arterial walls, leading to inflammatory destruction and progressive weakening of the vessel wall. Major predisposing factors include immunosuppression, advanced age, and underlying vascular disease [6,7].

We report a rare case of *Salmonella *Typhimurium, a non-typhoidal species that can cause invasive disease, presenting with the unusual coexistence of purulent pericarditis and a mycotic aortic aneurysm, a combination described in fewer cases [8-10]. The clinical course was further marked by severe complications, including constrictive pericarditis and cardiac tamponade. Given the exceptional rarity of this entity, this case highlights the challenges of acute management and underscores the need for sustained long-term follow-up.

## Case presentation

A 64-year-old woman with a long history of systemic lupus erythematosus, maintained on chronic corticosteroid therapy (prednisolone 20 mg/day), attended the emergency department after experiencing four days of fatigue, fever, and left-sided pleuritic chest pain radiating to the neck, which improved when leaning forward. She reported no recent epidemiological exposures, including recent travels or food poisoning. She denied gastrointestinal symptoms such as abdominal pain, nausea, vomiting, diarrhea, hematochezia, as well as respiratory or urinary symptoms. She also reported no rash, alopecia, anorexia, oral ulcers, arthralgia, or joint stiffness.

**Figure 1:**
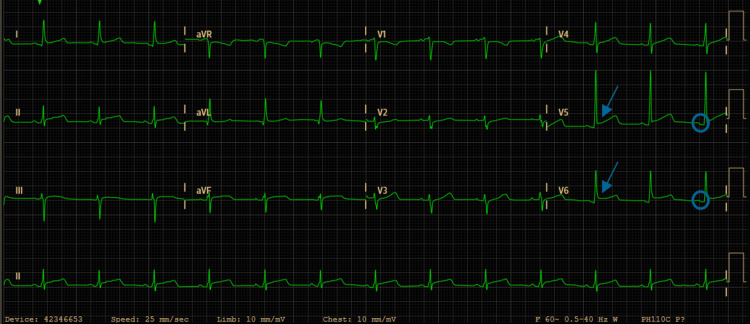
Electrocardiogram revealed ST-segment elevation with an upward-curving pattern (arrows) and concomitant PR-segment depression (circles) in leads V5–V6.

Initial laboratory testing showed normocytic, normochromic anemia (hemoglobin 10.1 g/dL) and elevated inflammatory markers, including leukocytosis (13,300/μL) and C-reactive protein (15.7 mg/dL). The electrocardiogram (ECG) showed widespread ST-segment elevation with an upward-curving pattern and concomitant PR-segment depression, most evident in leads V5-V6 (Figure 1). Based on these findings, the diagnosis of acute pericarditis was established, and she was discharged with an anti-inflammatory regimen consisting of ibuprofen 600 mg three times daily and colchicine 0.5 mg twice daily.

**Figure 2:**
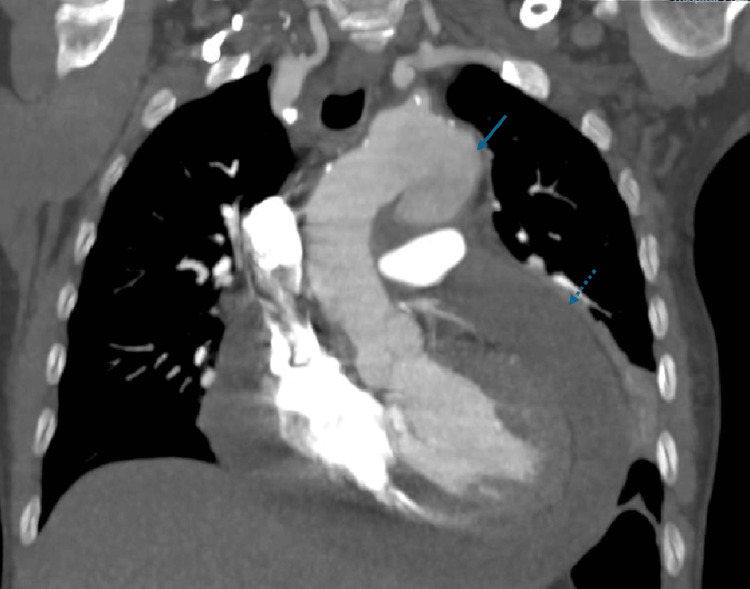
Thoracic CT angiography demonstrating a saccular aortic aneurysm (solid arrow) adjacent to the aortic arch (44×25×39mm) and a homogeneous pericardial effusion (dashed arrow) up to 3 cm in thickness. CT: computed tomography

Nine days after the initial assessment, she returned due to clinical deterioration, characterized by recurrent pleuritic chest pain and fever, along with new-onset dyspnea and orthopnea. On admission, she was febrile (38.3 °C) and tachypneic. Compared with the initial presentation, laboratory testing showed a further decline in hemoglobin (8.9 g/dL) and an increase in leukocytosis (15,600/μL), along with markedly elevated lactate dehydrogenase (700U/L), ferritin (2663 ng/mL), and erythrocyte sedimentation rate (78mm/h). Due to an elevated D-dimer level (4,565 µg/L), a thoracic computed tomography (CT) angiography was performed, ruling out pulmonary embolism but revealing a 4 cm saccular aneurysm adjacent to the aortic arch and a large homogeneous pericardial effusion measuring 3 cm in thickness (Figure 2).

**Figure 3:**
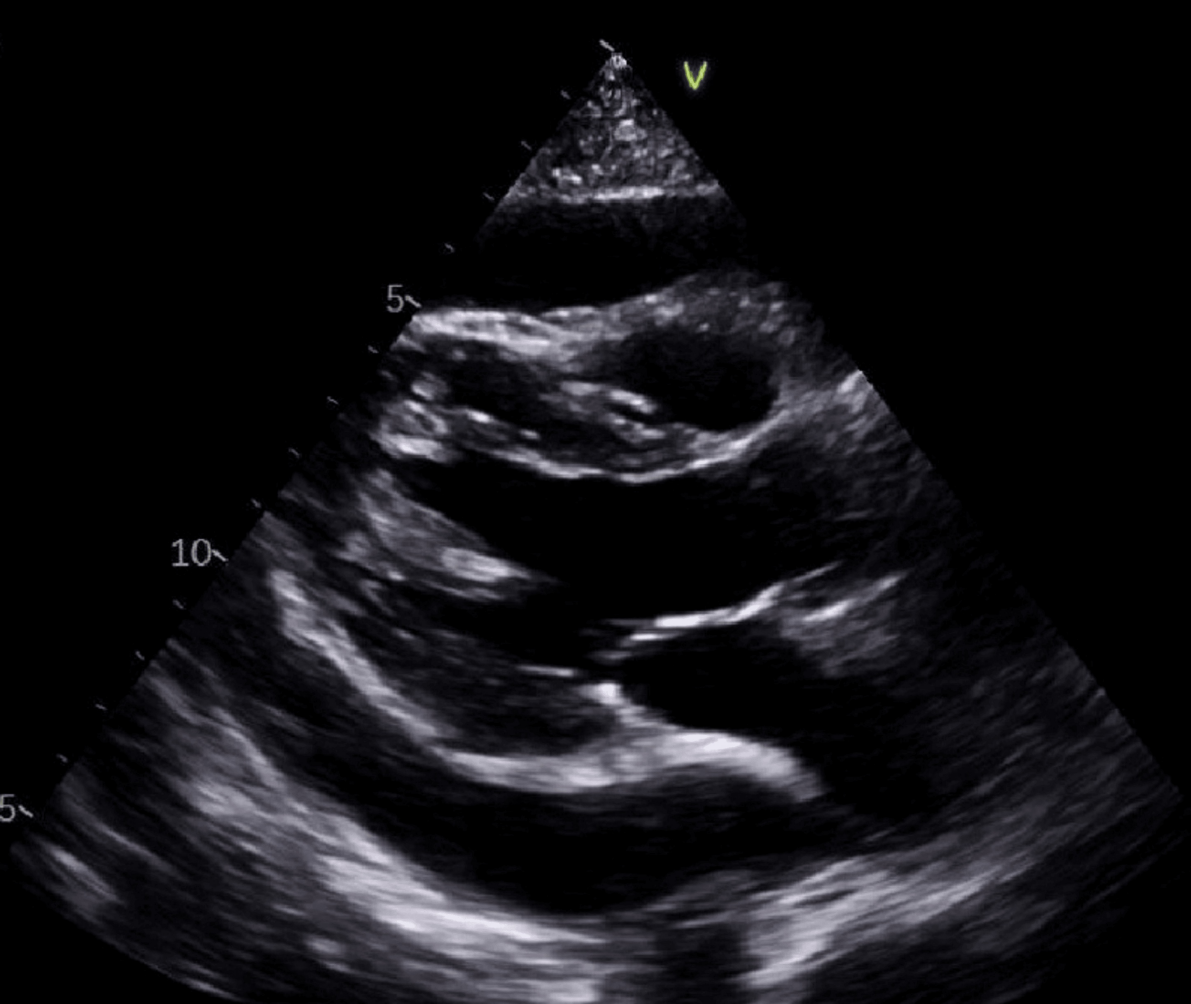
Parasternal long-axis view obtained prior to pericardiocentesis, showing a large pericardial effusion with a maximum measured dimension of 23 mm along the posterior wall.

**Figure 4:**
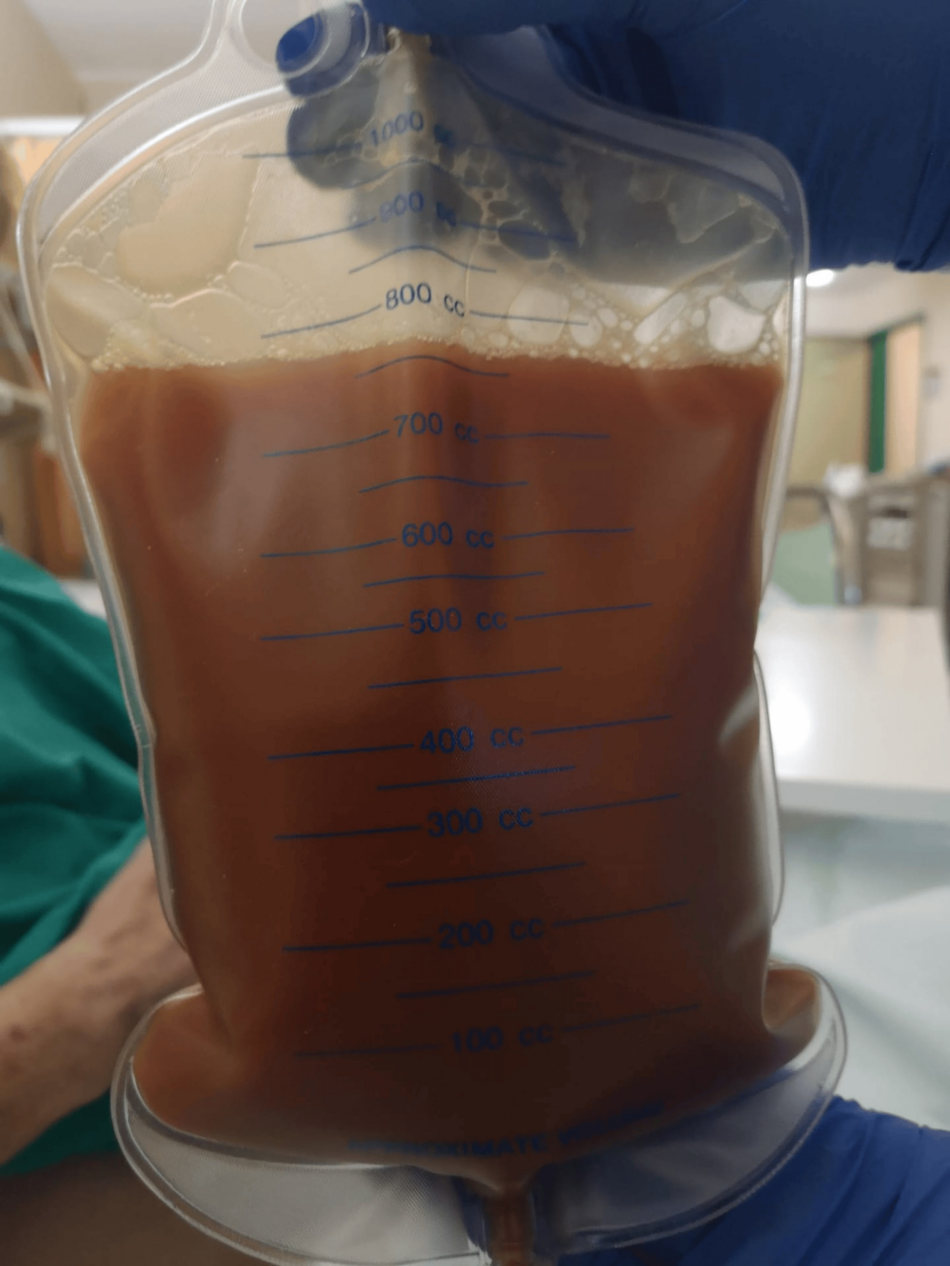
Purulent pericardial effusion obtained during pericardiocentesis.

Following admission, she developed arterial hypotension, and transthoracic echocardiography (TTE) showed a large pericardial effusion (Figure 3) and evidence of right ventricular diastolic collapse. Urgent pericardiocentesis was performed, draining 750 mL of purulent fluid (Figure 4). 

Biochemical analysis showed a low pericardial-to-serum glucose ratio and normal adenosine deaminase levels, and cytology identified 147,291/µL cells with neutrophilic predominance, supporting a bacterial etiology. Bacteriological culture of the pericardial fluid isolated *Salmonella *Typhimurium without detectable antimicrobial resistance. Blood cultures (aerobic and anaerobic) obtained before antibiotic therapy and at three and six days after treatment initiation were negative. Other microbiological studies, including urine and stool cultures, Ziehl-Neelsen staining, and Lowenstein-Jensen culture, were negative. Cytological examination of the pericardial fluid excluded malignancy.

Additional investigations were performed to exclude other infectious foci. Abdominal and pelvic CT angiography showed no evidence of additional aneurysms, and transesophageal echocardiography excluded infective endocarditis or other intracardiac foci. As part of the evaluation for alternative etiologies, autoimmune and endocrinologic testing, as well as serologic assays, including the Venereal Disease Research Laboratory test and human immunodeficiency virus testing, were negative. 

A diagnosis of invasive *Salmonella* Typhimurium infection causing purulent pericarditis and a mycotic thoracic aortic aneurysm was established, and intravenous ceftriaxone at a dose of two grams daily was started, supported by antimicrobial susceptibility testing showing a minimum inhibitory concentration (MIC) ≤1 mg/L. The evolution of the laboratory parameters throughout the clinical course is summarized in Table 1. 

**Table 1:** Evolution of laboratory parameters at presentation and during antibiotic therapy. ALP: alkaline phosphatase; ALT: alanine aminotransferase; APTT: activated partial thromboplastin time; AST: aspartate aminotransferase; BUN: blood urea nitrogen; CK: creatine kinase; CRP: C-reactive protein; ESR: erythrocyte sedimentation rate; GGT: gamma-glutamyl transferase; INR: international normalized ratio.

	Reference Values	First emergency department evaluation (Day 0)	Second emergency department evaluation (Day 9)	Fifth day of antibiotic therapy (Day 14)
					Hematology				
Hemoglobin (g/dL)	13.0-17.0	10.1	8.9	9.7
Haematocrit (%)	40-50	31.7	27.4	31
Mean corpuscular volume (fL)	78-96	93.8	89.5	90.8
Mean corpuscular hemoglobin (pg)	27-32	29.9	29.1	28.7
Leukocytes (x10^9^/L)	4.0-10.0	13.3	15.6	10.6
Neutrophils (x10^9^/L)	1.8-6.9	10.1	13.7	8.3
Lymphocytes (x10^9^/L)	1.2-3.3	1.3	0.7	1.4
Platelet count (x10^9^/L)	150-410	467	509	539
ESR (mm/h)	<13	-	78	-
Coagulation				
Prothrombin time (sec)	9.7-11.8	-	11.1	12.0
INR	<1.2	-	1.0	1.0
APTT (sec)	20.6-29.5	-	24.9	23.4
D-Dimer (µg/L)	<500	-	4565	
Biochemistry				
AST (U/L)	<40	-	43	38
ALT (U/L)	<40	-	72	50
ALP (U/L)	40-130	-	140	100
GGT (UI/L)	<60	-	374	100
Total bilirubin (mg/dL)	<1.2	-	0.7	0.4
Sodium (mmol/L)	136-145	139	137	143
Potassium (mmol/L)	3.5-5.1	5.1	4.6	3.9
Troponin T (mg/L)	<14	23	8	18
NT-proBNP (pg/mL)	<301	-	1250	-
CKI total (U/L)	26-192	24	23	43
Albumin (g/dL)	3.97-4.94	-	3.3	-
LDH (U/L)	132-225	-	700	415
Creatinine (mg/dL)	0.7-1.2	0.5	0.53	0.4
BUN (mg/dL)	<50	27.3	29	33
CRP (mg/dL)	<0.5	15.7	9.9	4.9

Despite clinical improvement with reduced pericardial drain output, resolution of fever from day seven of antibiotic therapy, and a decrease in inflammatory markers, serial transthoracic echocardiography demonstrated progressive features of constrictive pericarditis, including fibrinous pericardial content predominantly along the lateral and posterior walls, interventricular septal bounce, and a dilated inferior vena cava without respiratory collapse.

Due to the presence of constrictive pericarditis and a large infectious pseudoaneurysm of the aortic arch with a high risk of rupture, the patient underwent complete pericardiectomy and aortic arch replacement on hospital day 17. The procedure was performed without complications, and she was discharged after one month on a six-month regimen of oral ciprofloxacin 750 mg twice daily.

A summary of the clinical course is provided in Table 2.

**Table 2:** Clinical timeline outlining the main diagnostic findings, therapeutic interventions, and outcomes during the patient’s clinical course. CT, computed tomography; ECG, electrocardiogram; NSAIDs, non-steroidal anti-inflammatory drugs.

Day	Clinical events
Day 0	First emergency department presentation with symptoms and an ECG compatible with acute pericarditis. The patient was discharged on NSAIDs.
Day 9	Second emergency department presentation with clinical deterioration; CT angiography showed a large pericardial effusion and a saccular aortic arch aneurysm.
Day 9	Cardiac tamponade with hemodynamic compromise; urgent pericardiocentesis performed and ceftriaxone iniciated.
Days 10–16	Serial echocardiography demonstrating features of constrictive pericarditis.
Day 17	Complete pericardiectomy and aortic arch replacement.
Day 30	Discharged on long-term oral antibiotic therapy.

## Discussion

Non-typhoidal *Salmonella *(NTS) is the most frequent form of *Salmonella *infection worldwide and accounts for the majority of extra-intestinal manifestations [8-10]. Overall, 5-10% of infections progress to bacteremia, which may seed distant sites and result in focal complications such as meningitis, endocarditis, septic arthritis, and osteomyelitis [8]. Despite the absence of gastrointestinal symptoms, the present case illustrates two rare extra-intestinal complications of NTS infection occurring simultaneously in one patient, a mycotic aortic aneurysm and purulent pericarditis.

Mycotic aneurysms are uncommon but carry a substantial risk of rupture, exceeding that observed in atherosclerotic aneurysms [9]. Their incidence has declined with widespread antibiotic use, with *Staphylococcus aureus* remaining the leading pathogen [7]. *Salmonella *spp. exhibits a marked affinity for large arteries, enabling invasion of compromised vascular endothelium and contributing to infected aneurysm formation. Optimal management relies on prolonged targeted antimicrobial therapy combined with timely surgical intervention [9,10].

Purulent pericarditis is likewise a rare and life-threatening entity, with mortality rates of 15-40% despite appropriate treatment [1,5]. *Salmonella *is an uncommon causative pathogen, and in this case, the infection was further complicated by cardiac tamponade and constrictive pericarditis.

In this clinical presentation, chronic systemic corticosteroid therapy was the only identifiable risk factor for invasive *Salmonella *infection, highlighting the increased susceptibility of immunosuppressed individuals to organisms not typically associated with severe disease and emphasizing the importance of early consideration of non-idiopathic etiologies of pericarditis in these patients, along with close clinical monitoring.

## Conclusions

Purulent pericarditis and mycotic aortic aneurysm are both uncommon complications of non-typhoidal *Salmonella* infection. Their coexistence highlights the need to maintain a high index of suspicion for disseminated disease, particularly in immunosuppressed patients. This case underscores the importance of early diagnosis and aggressive management, including surgical intervention when clinically indicated, which is essential to achieving favorable clinical outcomes.
